# Whole-Genome Sequences of *Mycobacterium bovis* Strain MbURU-001, Isolated from Fresh Bovine Infected Samples

**DOI:** 10.1128/genomeA.01237-15

**Published:** 2015-11-05

**Authors:** Moira Lasserre, Luisa Berná, Gonzalo Greif, Florencia Díaz-Viraqué, Gregorio Iraola, Hugo Naya, Miguel Castro-Ramos, Arturo Juambeltz, Carlos Robello

**Affiliations:** a Unidad de Biología Molecular, Institut Pasteur de Montevideo, Montevideo, Uruguay; b Unidad de Bioinformática, Institut Pasteur de Montevideo, Uruguay; c Departamento de Bacteriología, División de Laboratorios Veterinarios (DI.LA.VE.), Montevideo, Uruguay; d Departamento de Bioquímica, Facultad de Medicina, Universidad de la República, Montevideo, Uruguay

## Abstract

Bovine tuberculosis in cattle has a high incidence in Uruguay, where it is considered a disease of national importance. We present the genome sequence of *Mycobacterium bovis* strain MbURU-001, isolated from pectoral lymph nodes of a bovine host from a cattle farm.

## GENOME ANNOUNCEMENT

*Mycobacterium bovis* is the etiological agent of bovine tuberculosis, an infectious, chronic disease considered one of the most important in dairy cattle. *M. bovis* has a wide host spectrum; besides cattle, it affects other mammal species, including humans (1) and many other wild animals, which act as a reservoir of the disease and hamper its eradication (2, 3). Moreover, bovine tuberculosis is a zoonotic risk of worldwide socioeconomic importance. In Uruguay, bovine tuberculosis is considered a disease of national importance that has shown a growing incidence in the last few years, and its high prevalence in Uruguay still does not have a clear epidemiological explanation. To gain better knowledge about the epidemiology of this disease in Uruguay and characterize its diversity, it is necessary to compare national strains to available sequences of this species to understand their biology. For these reasons, we sequenced and annotated the complete genome of *M. bovis* MbURU-001, a strain isolated from the pectoral lymph nodes of an infected cow from a cattle farm in Uruguay.

Sequencing of this strain was performed at the Institut Pasteur de Montevideo on an Illumina MiSeq platform from a paired-end library (2 × 75 cycles). Velvet (4) was used to perform a *de novo* assembly, followed by SPAdes (5). Next, CISA (6) software was used to integrate the results of the assemblies. The assembly was further improved using PAGIT toolkit (7) with the reference genome of *M. bovis* AF2122/97 (GenBank accession number NC_002945). The final assembly was automatically annotated with RAST (8). Single nucleotide polymorphisms (SNPs) versus *M. bovis* AF2122/97 were identified through VarScan (9) and the GATK pipeline (10), and snpEff (11) software was used to annotate the effects of these variations.

A total of 2,390,334 paired-end reads were assembled into 100 contigs, with an average coverage of 82-fold. This resulted in a genome size of ~4.30 Mb with a GC content of 65.5%. The genome of the *M. bovis* MbURU-001 strain contains 4,289 predicted coding sequences (CDSs), 1 rRNA operon, and 45 tRNA genes. A total of 596 SNPs and 46 indels were identified in this strain when it was compared to the reference**.** These SNPs affect 517 unique genes, of which 450 code for proteins. Fourteen genes were truncated by the introduction of stop codons, including one involved in lipid metabolism and a putative RNA methyltransferase; 275 genes had missense mutations, many involved in biosynthesis of secondary metabolites, antibiotics, and metabolism in different environments; and 12 genes were affected by frameshifts and involved in oxidative metabolism and translation. All 14 open reading frames (ORFs) from the PhiRv1 prophage were absent from this strain.

Further comparative genomics between this and other local and global sequenced strains may provide genotype-phenotype associations that might explain the diversity of phenotypes locally found, as well as provide more insights regarding *M. bovis* epidemiology.

### Nucleotide sequence accession numbers.

This whole-genome shotgun project has been deposited at DDBJ/EMBL/GenBank under the accession number LFGY00000000. The version described in this paper is version LFGY01000000 and consists of sequences LFGY01000001 to LFGY01000100.
